# Immunologic Response to SARS-CoV-2 Vaccination in Pediatric Kidney Transplant Recipients: A Systematic Review and Meta-Analysis

**DOI:** 10.3390/vaccines11061080

**Published:** 2023-06-09

**Authors:** Elpida Emmanouilidou-Fotoulaki, Vasiliki Karava, John Dotis, Antonia Kondou, Nikoleta Printza

**Affiliations:** Pediatric Nephrology Unit, 1st Department of Pediatrics, Aristotle University of Thessaloniki, 54642 Thessaloniki, Greece

**Keywords:** COVID-19, vaccine, kidney, transplantation, pediatric, risk factors

## Abstract

The pediatric population is at a lower risk of severe SARS-CoV-2 infection compared to adults. Nevertheless, immunosuppression in pediatric and adolescent kidney transplant recipients (KTRs) increases their hazard compared to the general population. This systematic review evaluates the efficacy of SARS-CoV-2 vaccines and determines the risk factors of no seroconversion in this population. PubMed-MEDLINE databases were searched for cohort studies. A meta-analysis was performed using fixed and random effect models. In total, seven studies including 254 patients were further analyzed. The random effect model demonstrated a 63% seroconversion rate (95% CI 0.5, 0.76) following a two-dose schedule, which increased to 85% (95% CI 0.76, 0.93) after the third dose administration. Seropositivity was lower in patients under mycophenolate mofetil compared to azathioprine (OR 0.09, 95% CI 0.02, 0.43). Rituximab administration decreased the seroconversion rate (OR 0.12, 95% CI 0.03, 0.43). The glomerular filtration rate (GFR) was 9.25 mL/min/1.73 m^2^ lower (95% CI 16.37, 2.13) in patients with no seroconversion. The seroconversion rate was lower in vaccinated compared to infected patients (OR 0.13, 95% CI 0.02, 0.72). In conclusion, vaccination against SARS-CoV-2 in pediatric and adolescent KTRs elicits a humoral response, and a third dose is advised. Previous rituximab administration, antimetabolite therapy with mycophenolate mofetil and lower GFR reduce the likelihood for seroconversion.

## 1. Introduction

Since the first successful pediatric kidney transplantation in 1959, growing knowledge on pediatric pretransplant preparation and improvement of surgical techniques and postoperative care have contributed to the impressive improvement of both patient and graft survival [1]. Consequently, pediatric kidney recipients’ outcomes are now reported to be equal to adults’, and children often experience even better long-term graft survival [2]. Thus, despite ongoing challenges, kidney transplantation is the optimal treatment of end-stage kidney disease in the pediatric population.

Nowadays, more than 1300 pediatric kidney transplantations are performed worldwide each year [1], and registries report an increasing prevalence among children. More specifically in Europe, the prevalence of pediatric kidney transplantation increased 1.5% annually from 2010 to 2016 with the annual overall number of performed kidney transplantations varying between 3.1 and 3.9 per million age-related population [3]. Based on the Annual Data Report in the USA, the total number reached the highest point in 2021 with a total of 820 pediatric kidney transplants, a trend driven by the growth in deceased donors [4].

The COVID-19 pandemic, announced as a public health emergency in March 2020, has affected adult kidney transplant recipients (KTRs) in many ways, including waitlist removals, living and deceased donor kidney transplantations and mortality rates [4]. First, the waitlist for kidney transplantation was raised, leading to augmentation of COVID-19 infection and overall mortality among the waitlisted patients [5,6]. Moreover, deceased donor kidney transplantations slightly increased [4]. Furthermore, long-term immunosuppressive therapy coupled with various coexisting comorbidities, such as arterial hypertension, commonly observed in KTRs, increased the risk of severe COVID-19 infection and hospitalization in this vulnerable population, which experienced a higher mortality rate compared to the general population and dialysis patients on the waitlist [5,6,7,8].

Children and adolescents exhibit a broad range of clinical manifestations from SARS-CoV-2 infection, with the majority having asymptomatic disease or experiencing minimal and mild symptoms [9]. Better local airway and thymic immune responses as well as an abundance of cross-reactive T cells and antibodies present in the pediatric compared to adult population have been suggested as contributive factors explaining this condition [9]. Nevertheless, a severe course with respiratory failure and pediatric inflammatory multisystem syndrome, defined as a multiorgan disease syndrome occurring 2–6 weeks after COVID-19 infection, has also been described [9]. As in the adult population, the COVID-19 pandemic challenged pediatric kidney transplantation programs to provide efficient and timely care. In the early phase of the pandemic, living donor kidney transplantations were critically reduced and deceased donor kidney transplantations were prioritized for those of highest need occasionally allowing COVID-19 positive donors or recipients [10,11]. Outcomes of SARS-CoV-2 infection did not differ between pediatric KTRs and the general pediatric population [11,12]. Nevertheless, both pediatric KTRs and pediatric dialysis patients exhibited a high risk of COVID-19 infection, with an incidence rate of 9.2% and 9.3% respectively, according to a recent multicenter study [13].

COVID-19 vaccine distribution guidelines have prioritized KTRs [14]. Literature however supports that humoral immunity in solid organ transplant recipients is impaired, based on evidence against previously developed vaccines, such as hepatitis B and influenza vaccines [15]. For instance, although a high or booster dose of the influenza vaccine is currently not recommended during the same season in KTRs, patients with a post-vaccination hepatitis B antibody titer less than 10 U/mL should be revaccinated [15]. A lower immunogenic response affecting both humoral and cellular responses is also described after SARS-CoV-2 vaccination in adult KTRs, suggesting that they are vulnerable to future infections [16]. For instance, according to Grupper et al., SARS-CoV2-IgG antibodies after a two-dose mRNA vaccination regimen were reported in 100% of 25 healthy controls but only in 37.5% of 136 KTRs [16]. As in the adult population, inactivated vaccines are considered safe in pediatric KTRs, but their efficacy is put into question, due to the recipient’s diminished immunological response and supplementary doses may be required [17]. The humoral response to SARS-CoV-2 vaccines has been addressed in some studies on children and adolescents KTRs. To that end, we conducted a systematic review and meta-analysis to evaluate the immunogenic response of SARS-CoV-2 vaccines in pediatric and adolescent KTRs.

## 2. Materials and Methods

This systematic review and meta-analysis was conducted according to the Preferred Reporting Items for Systematic Reviews and Meta-Analyses (PRISMA) guidelines (PRISMA Checklist, Table S1). PubMed-MEDLINE was searched for original articles reporting the efficacy of the SARS-CoV-2 vaccine in pediatric and adolescent KTRs and the last search was conducted in December 2022. To that end, the following search term was used:

((COVID-19) OR (SARS-CoV-2) OR (coronavirus)) AND ((vaccine) OR (vaccination) OR (immune)) AND ((kidney transplant) OR (renal transplant)) AND ((child*) OR (adolesc*) OR (pediatr*)).

The references of the selected articles were further screened to search for potentially relevant articles. The literature review, data extraction and study quality assessment were independently performed by two reviewers, and any disagreement was resolved by consensus.

### 2.1. Eligibility Criteria

Studies investigating the immunogenicity of the COVID-19 mRNA vaccination in pediatric and adolescent KTRs were eligible for inclusion if they met the following criteria: (1) population: pediatric and adolescent KTRs; (2) intervention: COVID-19 mRNA vaccination; (3) study design: retrospective and prospective cohort and case-control studies; (4) outcome: seroconversion rate using anti-SARS-CoV-2 spike IgG after the second and/or the third dose of COVID-19 mRNA vaccines. The exclusion criteria were the following: (1) adult studies or unclear participants’ age; (2) case reports; (3) articles not written in English; and (4) studies with overlapping participants.

### 2.2. Data Extraction

The following data were extracted from the included articles: author, date of publication, country of origin, study design, study sample size, inclusion and exclusion criteria, mean participants’ age, kidney transplant duration, presence or absence of prior COVID-19 infection, number of received vaccine doses, type of vaccination, seroconversion rate after the second and/or third vaccine dose, post-vaccination time of SARS-CoV-2 IgG titer assessment and risk factors of no seroconversion. When feasible, data on the neutralization activity of the antibodies obtained after the vaccine regimen, adverse events of vaccination, and characteristics and seroconversion rate of healthy controls in case-control studies were collected. All study characteristics were tabulated and reviewed by the two reviewers.

### 2.3. Statistical Analysis

Review Manager 5.4 for Windows and R version 4.1.2 for Windows were utilized for the meta-analysis. The I^2^ test was used in order to measure the heterogeneity of the studies, where I^2^ ≥ 50% indicated high heterogeneity. A fixed model overall pool estimate was employed if the heterogeneity of the studies was below 40% and a random effect model was employed in the case of heterogeneity above 40%. A forest plot was applied for illustration of the combined estimated outcomes from the different studies. Effect measures were calculated as proportions for seroconversion after the second and third doses and the Odds Ratio (OR) was calculated for all other results. Publication bias was assessed with Egger’s test, although the power of the test is low in small samples. The quality review of the bias of the cohort studies was performed using the Newcastle-Ottawa Scale. Studies with 3 stars on selection domain, 1 or 2 stars in comparability domain and 2 or 3 stars in outcome/exposure domain were considered good quality. Sensitivity analysis, including only good quality studies, was performed when more than three good quality studies were available for meta-analysis. The *p*-value threshold for statistical significance was set at 0.05.

## 3. Results

### 3.1. Study Selection

**Figure 1:**
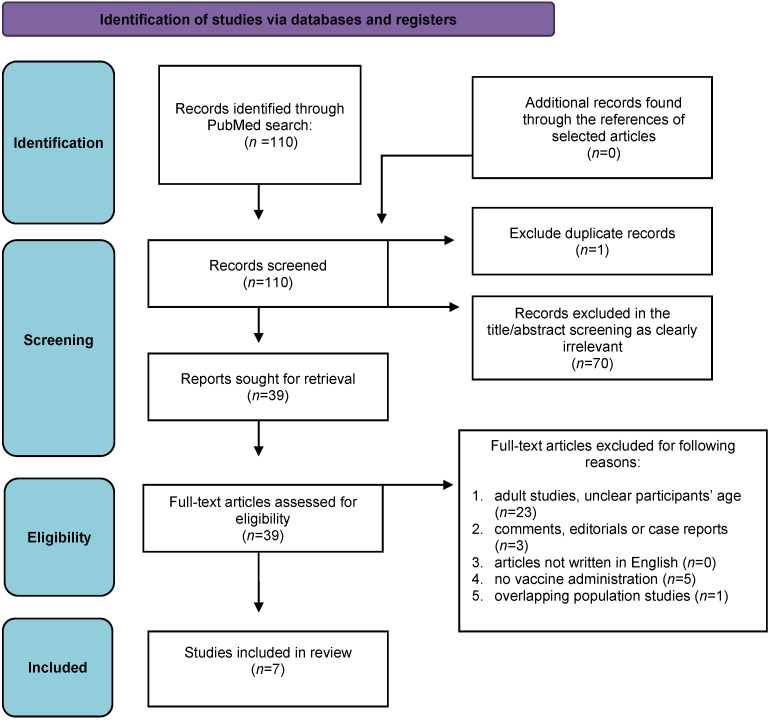
The PRISMA flow diagram of the study.

The study selection flowchart is presented in Figure 1. The literature search resulted in 110 studies of which 70 were considered irrelevant based on abstract or title screening and one was a duplicate. Full-text access was available for the remaining 39 studies, and further 32 studies were removed according to the exclusion criteria. Therefore, seven studies were finally eligible for the meta-analysis.

### 3.2. Characteristics of the Included Studies

The included studies characteristics are provided in Table 1 [18,19,20,21,22,23,24]. All seven selected studies included 254 pediatric and adolescent KTRs, who were naïve to COVID-19 infection, based on prior to vaccination SARS-CoV-2 IgG titers or patient medical history, and who received two doses of the SARS-CoV-2 mRNA vaccine. A total of 95 patients received a third dose. Five studies incorporated a control group and almost all participants were administered the BNT162b2 COVID-19 mRNA vaccine. Among the included studies, two were conducted in Germany, one in USA, one in Italy, one in Turkey, one in Australia and one in Israel. Among the studies, five were retrospective and two prospective.

The quality assessment of the studies is illustrated in Table 2. Of note, the absence of SARS-CoV-2 IgG at study initiation was obtained in three studies, which were considered good quality [21,22,23]. In the rest of the studies, the absence of COVID-19 infection was mainly based on the patient’s medical history. The length and adequacy of follow-up were satisfactory in all the included studies. In most studies, the statistical analysis was controlled for age and other factors, including the type of immunosuppressive therapy.

### 3.3. Seroconversion after the Second and the Third Dose of COVID-19 mRNA Vaccine

**Figure 2:**
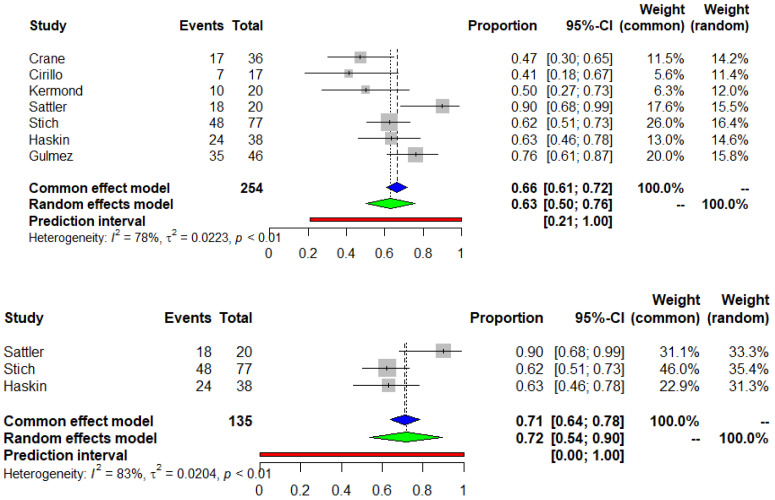
Meta-analysis of the seroconversion rate after the second dose of COVID-19 mRNA vaccine in pediatric and adolescent COVID-19 naïve kidney transplant recipients including all studies and only good quality studies. Blue and green triangle correspond to the overall proportion according to the common effect and random effect model respectively.

**Figure 3:**
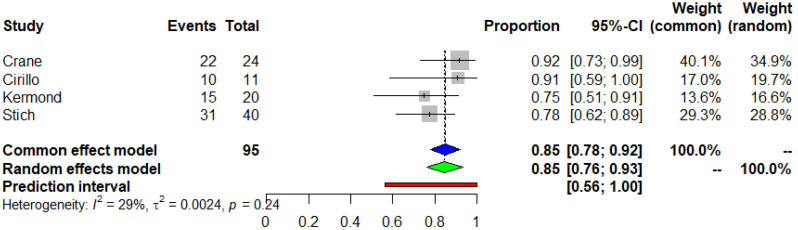
Meta-analysis of the seroconversion rate after the third dose of COVID-19 mRNA vaccine in pediatric and adolescent COVID-19 naïve kidney transplant recipients. Blue and green triangle correspond to the overall proportion according to the common effect and random effect model respectively.

**Figure 4:**
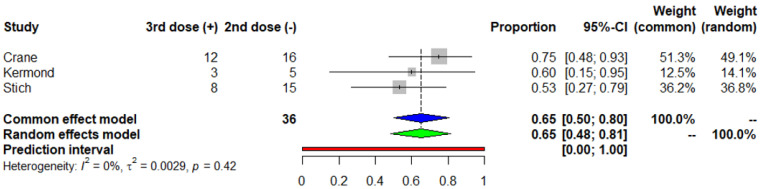
Meta-analysis of the seroconversion rate after the third dose of COVID-19 mRNA vaccine in pediatric and adolescent COVID-19 naïve kidney transplant recipients who were non-responders to the second dose. Blue and green triangle correspond to the overall proportion according to the common effect and random effect model respectively.

The mean participant age (SD) was 16.7 (2.6) years. The meta-analysis of seven studies (I^2^ = 78%) revealed that 63% of the pediatric and adolescent COVID-19 naïve KTRs developed positive seroconversion after a standard two-dose SARS-CoV-2 vaccine regimen (95% CI 0.5, 0.76) (Egger’s test *p* = 0.178) (Figure 2). In a further analysis that included only good quality studies, the seroconversion rate was 72% (95% CI 0.54, 0.90). Moreover, 95 patients, included in four studies, were administered a third dose and the humoral immune response was positive in 85% of the patients (95% CI 0.76, 0.93) (Egger’s test *p* = 0.701) (Figure 3). Three of the included studies were conducted on 36 seronegative patients after the second dose. An immune response with positive SARS-CoV-2 IgG titers was observed in 23 of the previous non-responders (65%, 95%CI 0.48, 0.81) after a third vaccine dose administration (Egger’s test *p* = 0.569) (Figure 4). The median time between the second vaccination and assessment of immune response was 38.9 days.

### 3.4. Risk Factors of no Seroconversion after the Second Dose of the COVID-19 mRNA Vaccine

**Figure 5:**
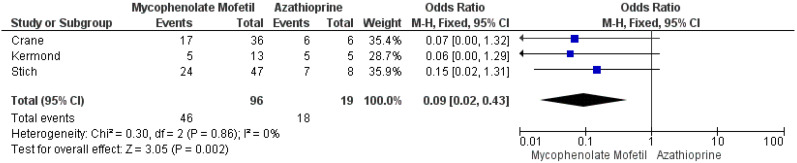
Meta-analysis of the seropositivity after the second dose of the COVID-19 mRNA vaccine in pediatric and adolescent kidney transplant recipients under mycophenolate mofetil or azathioprine.

**Figure 6:**
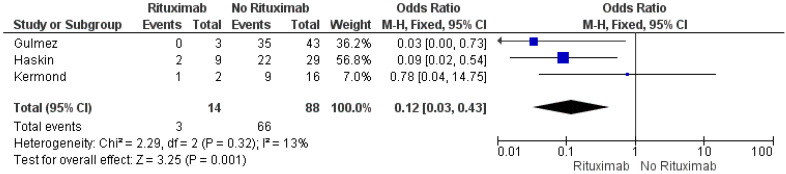
Meta-analysis of the seropositivity after the second dose of the COVID-19 mRNA vaccine in pediatric and adolescent kidney transplant recipients having received or not received Rituximab therapy.

**Figure 7:**
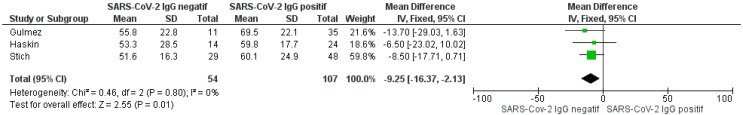
Meta-analysis of the glomerular filtration rate (GFR) expressed in ml/min/1.73 m^2^ in pediatric and adolescent kidney transplant recipients with or without SARS-CoV-2 IgG positive titers after the second dose of the COVID-19 mRNA vaccine.

Multiple risk factors of no seroconversion were reported. Specifically, Crane et found that a younger age (<12 years) was significantly associated with higher seroconversion rate [18], while Stich et al. [23] and Gulmez et al. [24] observed that female sex and longer transplant duration were independent risk factors for non-seropositivity. Three studies compared seroconversion rate between the patients under mycophenolate mofetil (MMF) and azathioprine. According to the meta-analysis results on 96 participants (I^2^ = 0%), the seroconversion rate was 10-fold higher in KTRs on azathioprine compared to those on MMF (Figure 5). Moreover, based on the data from three studies (I^2^ = 13%), KTRs were 88% less likely to develop seroconversion if they had previously received Rituximab therapy (OR 0.12, 95% CI 0.03, 0.43) (Figure 6). Of note, MMF and prednisone dose were associated with a lower seroconversion rate according to the results of Cirillo et al. [19] and Kermond et al. [20] respectively. Furthermore, among 54 patients with no humoral response, the glomerular filtration rate (GFR) was 9.25 mL/min/1.73 m^2^ lower than that of 107 KTRs who developed SARS-CoV-2 IgG titers after the second dose of the COVID-19 mRNA vaccine (95% CI −16.37, −2.13) (Figure 7).

### 3.5. Neutralization Activity

Among the included studies, three investigated serum neutralizing activity against SARS-CoV-2. Specifically, in the Stich et al. study, 22.2% of KTRs presented functional neutralizing activity against the omicron (BA.1) variant using a live virus neutralization assay [23]. In the Sattler et al. study, 75% of KTRs presented adequate virus neutralizing capacity using a blocking ELISA (sVNT kit, GenScript) mimicking the virus neutralization process [21]. Finally, in the Gulmez et al. study, 54.3% of KTRs presented adequate neutralizing antibody response, assessed as percent inhibition (%IH) [24].

### 3.6. Adverse Events and Renal Outcomes

With respect to adverse events, no significant safety concerns were reported. Crane et al. [18] reported no serious adverse events after vaccination and Haskin et al. [22] described mild to moderate adverse reactions at the injection site such as pain (65%), redness (16%) and swelling (11%) and systemic symptoms, such as fatigue (41%), headache (35%), chills (8%), nausea (16%), diarrhea (5%), muscle pain (32%) and joint pain (5%). The effects on renal outcome were assessed in two studies. In the Haskin et al. study, one patient developed borderline cellular rejection two weeks post vaccination [22], while acute rejection or diagnosis of de novo glomerular disease was not recorded within the six-month period post vaccination in the Crane et al. study [18]. In the Haskin et al. study, pre-vaccination eGFR was slightly higher (56.6 ± 21.5 versus 57.4 ± 22.1 mL/min/1.73 m^2^; *p* < 0.001) and mean serum creatinine levels were lower (1.48 ± 0.94 versus 1.43 ± 0.93 mg/dL, *p* < 0.001) when compared to post-vaccine levels, although the difference did not reach statistical significance [22].

### 3.7. Comparison of Immune Response in KTRs and Controls

The immune response in healthy individuals was assessed in two studies. The data on 29 healthy individuals demonstrated significantly increased IgG, IgA and neutralization capacity levels when compared to KTRs [21,24], and whereas spike specific CD4+ T cell frequencies were similar in both groups, cytokine production and memory differentiation were significantly impaired in KTRs [21]. Patients with chronic kidney disease had higher serum levels of anti-SARS-CoV-2 IgG [23,24] neutralizing antibody activity and Interferon-Gamma Release Assay (IGRA) titers [24]. Stich et al. described a statistically significant difference of humoral immune response rates between KTRs (62.3%), patients with CKD on immunosuppressive medication (80.8%) and patients with CKD without immunosuppressive medication (95%) [23]. When compared to CKD without immunosuppressant therapy, anti-S1 Receptor Binding Domain (RBD) IgG level was ninefold lower (*p* < 0.001) in KTRs (117 [IQR 0–769] BAU/mL versus 1046 [IQR 470–2735] BAU/mL) [23].

### 3.8. Comparison of Serologic Response between Vaccinated and Naturally Infected Patients

**Figure 8:**
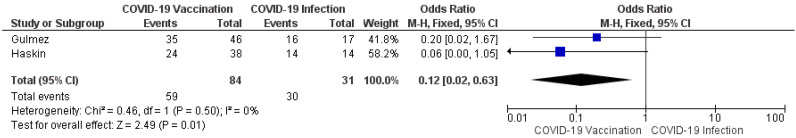
Meta-analysis of the seropositivity in pediatric and adolescent kidney transplant recipients with COVID-19 infection or COVID-19 mRNA vaccination.

The results on 84 COVID-19 vaccinated and 31 COVID-19 infected patients demonstrate that the serologic response is 87% less likely (OR 0.13, 95% CI 0.02, 0.72) after vaccination compared to natural infection (Figure 8). In addition, anti-SARS-CoV-2 IgG titers were found to be higher in COVID-19 infected compared to vaccinated patients with Haskin et al. demonstrating an almost 30-fold higher median titer level (2782 AU/mL (IQR: 1908–11 000) versus 100.3 AU/mL (IQR: 4.7–1744), *p* = 0.0008) [22].

## 4. Discussion

This systematic review study on pediatric and adolescent KTRs demonstrated a 63% immunologic response rate after a two-dose vaccination for SARS-CoV-2. The proportion improved to 85% after a third dose with a seroconversion of 65% of previous non-responders. Administration of MMF and Rituximab as part of immunosuppression therapy and lower GFR were associated with a lower humoral response. An enhanced serologic response was reported in naturally infected individuals compared to vaccinated ones, and no severe adverse events were documented after immunization with the exception of a borderline cellular rejection occurring in one patient two weeks post vaccination.

The positive humoral immune response reported in our meta-analysis is relatively higher compared to that in adult KTRs. Recent literature indicates inadequate seroconversion in adult KTRs with seropositivity following a standard immunization course varying from 30% to 51.4% among different studies [25,26,27,28]. In addition, in the adult population, older age and longer dialysis vintage before kidney transplantation were indicated as risk factors of a lower seroconversion rate [29]. Hence, young age is associated with enhanced immunogenicity of SARS-CoV-2 vaccines and the latter could be taken into consideration when prioritization of a booster vaccination is considered. Whereas our results are encouraging, a recent review indicates that the efficacy of vaccination among healthy children and adolescents ranges from 88% to 100% [30], which is significantly higher than that reported in our meta-analysis, indicating an impaired immunity in young KTRs compared to healthy controls. As current literature supports, suboptimal vaccine efficacy is anticipated in patients with immunosuppression and in particular in solid organ transplant recipients [31].

Data regarding neutralizing activity against SARS-CoV-2 variants are limited in both adult and pediatric KTRs. Benning et al. remarked that antispike 1, antireceptor-binding domain, and surrogate neutralizing antibodies were detected in 30%, 27%, and 24% of adult KTRs respectively, while neutralization against B.1.351 and B.1.617.2 was observed in 64% and 67% of adult KTRs, respectively [25]. In this systematic review, neutralizing activity was assessed in three studies, analyzed with different methods, and ranged from 22.2% to 75% [21,23,24]. Interestingly, Grupper et al. observed that the seroconversion rate was significantly lower in KTRs compared to those vaccinated before kidney transplantation [29]. These findings are in accordance with the results from the two studies, included in this systematic review, where seroconversion rate was higher in patients with CKD compared to KTRs [23,24].

The type of immunosuppressive treatment has emerged as an important factor contributing to seroconversion. The use of antimetabolites is associated with reduced immunogenicity in solid organ transplant recipients [32], and prior Rituximab induced B-cell depletion has been described as a strong predictor of seroconversion failure not only after SARS-CoV-2 but also after the influenza vaccine [33,34]. A temporary hold of treatment substantially increases immunogenicity [35,36], but data on this topic are limited. A fourth dose of the SARS-CoV-2 vaccine during a short-term (five weeks) MMF-based treatment withdrawal in KTRs who were seronegative after triple vaccination resulted in a significant increase in the humoral response without any evidence of renal impairment, emphasizing withhold safety [37]. The same results were reported after a five-week mycophenolic acid (MPA) hold (adding 5 mg prednisolone equivalent in case of steroid-free treatment), whereas no significant change in serological response rate was remarked after MPA reduction [38]. Regarding immunosuppressed participants other than KTRs, the American College of Rheumatology recently recommended withholding MMF 1-week postvaccination [36], while an observational trial concluded that 28.5% of patients with rheumatic conditions paused their medication before or during vaccination even without medical consulting in advance [39]. The absence of data on pediatric KTRs suggest that temporary withhold should be sought with caution. Nevertheless, T-cell immunity is preserved in these patients and vaccine induced T-cell immunity may provide sufficient protection given than successful recovery of COVID-19 has been reported in patients with agammaglobulinemia without developing humoral response [40,41].

Lower seroconversion rates and reduced magnitude of antibody response after vaccination compared to natural infection were observed in this systematic review and are consistent with literature data regarding other vaccines in general population. For instance, not only were the antibody titers generated by vaccination lower than those induced by natural mumps infection [42], but also the magnitude and duration of cell-mediated immunity were greater after rubella natural infection [43]. Finally, the safety and tolerability of the COVID-19 vaccination that emerged from this study are in accordance with previous results from adult KTRs [28]. Ślizień et al. reported no serious adverse events after COVID-19 mRNA vaccination in 300 KTRs, while fatigue, headache and myalgia were the most common systemic reactions [28]. Interestingly, in the same study, systemic reactions were most frequently observed in younger patients [28].

Although our literature review and meta-analysis was undertaken in a systematic way, our study has some limitations. Protocol registration was eliminated. Nevertheless, the quality of the included studies was thoroughly assessed by two independent reviewers. Moreover, limited literature was available, and both retrospective and prospective studies with fair and good quality were included possibly reducing the level of evidence. Although the methods used to assess response rates varied among studies, the heterogeneity of the studies in most cases was low, therefore the fixed effect model was preferred. This could be explained by the wide, overlapping confidence intervals and the relatively small number of included studies. In cases where the heterogeneity was high, the random effect approach was used in order to take into consideration the between-study variance.

## 5. Conclusions

In conclusion, despite the limited number of studies and participants, according to our results, seropositivity after a two-dose vaccine regimen against SARS-CoV-2 in pediatric and adolescent KTRs is suboptimal, and therefore, a third dose is advised. Reassuringly, our data confirm that third dose should be recommended to optimize seroconversion, but further studies should focus on strategies to enhance immunogenicity. Previous Rituximab administration, antimetabolite therapy with MMF and lower GFR are risk factors of non-seroconversion emphasizing the need for a multifaceted approach regarding the timing of vaccination and suggesting patient-centered care. Longer and larger follow-up studies are required in order to determine immunogenicity duration and types of vaccine-induced protection.

## Figures and Tables

**Table 1:** Characteristics of the included studies.

First Author	Study Design	Patient Age (Years)	Time Post KT (Years)	Patients				Characteristics of Controls	Type of Vaccine
				2nd Dose		3rd Dose			
				N	Time of IgG Measurement (Days)	N	Time of IgG Measurement (Days)		
Crane [18]	Retrospective and prospective cohort	Median (IQR):18 (15–20)	Median (IQR): 5 (2–9)	43 (7 with prior infection)	Median (IQR):56 (30–85)	26 (2 with prior infection, 16 not responded to 2nd dose)	Median (IQR):39 (28–59)	-	40 (93%) patients received BNT162b2, 2 (5%) mRNA1273, and 1 patient a mixed vaccine series
Cirillo [19]	Retrospective cohort and case-control	Mean (SD):19(2)	NA	18 (1 with prior infection)	NA	12 (1 with prior infection)	NA	Healthy controls	mRNA
Kermond [20]	Retrospective cohort	Median (IQR):15 (12–16)	Median (range): 11 (2 months–14 years)	20	Median (IQR): 38.5 (32.5–57.5)	20 (5 not responded to 2nd dose)	Median (IQR): 44 (40–52)	-	BNT162b2
Sattler [21]	Retrospective cohort and case-control	Mean (SD):14.17 (1.31)	Mean (SD): 7 (4.1)	20	Mean (SD): 39.30 (11.06)	-	-	13 healthy controls	BNT162b2
Haskin [22]	Prospective cohort and case-control	Mean (SD):18(3)	Mean (SD): 7.3 (5.6)	38	Median (IQR): 37 (20.5–53)	-	-	14 KTRs with prior COVID-19 infection	BNT162b2
Stich [23]	Retrospective cohort and case-control	Median (range): 14.1 (5–30)	NA	77	Median (IQR): 34 (22–63)	40 (15 not responded to 2nd dose)	NA	26 CKD patients with IS 20 CKD patients without IS	BNT162b2
Gulmez [24]	Prospective cohort and case control	Mean (SD): 15.9(2.86)	NA	46	Median (range): 8 weeks (7–14 weeks)	-	-	19 KTRs with prior COVID-19 infection 19 patients on dialysis 19 healthy controls	BNT162b2

CKD: chronic kidney disease, IS: immunosuppression, KT: kidney transplantation, KTRs: kidney transplant recipients, NA: not available.

**Table 2:** Quality assessment of the included studies using the Newcastle-Ottawa Scale (NOS).

First Authors	Selection			Compatibility	Outcome			Overall NOS Score
	Representativeness of the Exposed Cohort	Ascertainment of Exposure	Absent Outcome at Study Initiation		Assessment of Outcome	Follow-Up Duration	Adequacy of Follow-Up	
Crane [18]	*	*		**	*	*	*	7
Cirillo [19]	*	*			*	*	*	5
Kermond [20]	*	*		**	*	*	*	7
Sattler [21]	*	*	*	**	*	*	*	8
Haskin [22]	*	*	*	**	*	*	*	8
Stich [23]	*	*	*	**	*	*	*	8
Gulmez [24]	*	*		**	*	*	*	7

* Each asterisk corresponds to 1 star.

## Data Availability

The data presented in this study are available on request from the corresponding author.

## Supplementary Materials

The following supporting information can be downloaded at: https://www.mdpi.com/article/10.3390/vaccines11061080/s1, Table S1: PRISMA Checklist [44].

## References

[1] Oomen L., Bootsma-Robroeks C., Cornelissen E., de Wall L., Feitz W. (2022). Pearls and Pitfalls in Pediatric Kidney Transplantation After 5 Decades. Front Pediatr..

[2] Verghese P. (2017). Pediatric kidney transplantation: A historical review. Pediatr. Res..

[3] Bonthuis M., Vidal E., Bjerre A., Aydoğ Ö., Baiko S., Garneata L., Guzzo I., Heaf J.G., Jahnukainen T., Lilien M. (2021). Ten-year trends in epidemiology and outcomes of pediatric kidney replace-ment therapy in Europe: Data from the ESPN/ERA-EDTA Registry. Pediatr. Nephrol..

[4] Lentine K.L., Smith J.M., Miller J.M., Bradbrook K., Larkin L., Weiss S., Handarova D.K., Temple K., Israni A.K., Snyder J.J. (2023). OPTN/SRTR 2021 Annual Data Report: Kidney. Am. J. Transplant..

[5] Craig-Schapiro R., Salinas T., Lubetzky M., Abel B.T., Sultan S., Lee J.R., Kapur S., Aull M.J., Dadhania D.M. (2021). COVID-19 outcomes in patients waitlisted for kidney transplantation and kidney transplant recipients. Am. J. Transplant..

[6] Clarke C., Lucisano G., Prendecki M., Gleeson S., Martin P., Ali M., McAdoo S.P., Lightstone L., Ashby D., Charif R. (2021). Informing the Risk of Kidney Transplantation Versus Remaining on the Waitlist in the Coronavirus Disease 2019 Era. Kidney Int. Rep..

[7] Hilbrands L.B., Duivenvoorden R., Vart P., Franssen C.F.M., Hemmelder M.H., Jager K.J., Kieneker L.M., Noordzij M., Pena M.J., Vries H. (2020). COVID-19-related mortality in kidney transplant and dialysis patients: Results of the ERACODA collaboration. Nephrol. Dial. Transplant..

[8] Gagliardi I., Patella G., Michael A., Serra R., Provenzano M., Andreucci M. (2020). COVID-19 and the Kidney: From Epidemiology to Clinical Practice. J. Clin. Med..

[9] Brodin P. (2022). SARS-CoV-2 infections in children: Understanding diverse outcomes. Immunity.

[10] Hogan J., Kwon T., Paye-Jaouen A., Fait C., Cointe A., Baudouin V. (2021). Kidney Transplantation in a COVID-19-positive Pediatric Recipient. Transplantation.

[11] Teoh C.W., Gaudreault-Tremblay M.M., Blydt-Hansen T.D., Goldberg A., Arora S., Feber J., Langlois V., Ruhl M., Phan V., Morgan C. (2020). Management of Pediatric Kidney Transplant Patients During the COVID-19 Pandemic: Guidance From the Canadian Society of Transplantation Pediatric Group. Can. J. Kidney Health Dis..

[12] Marlais M., Wlodkowski T., Vivarelli M., Pape L., Tönshoff B., Schaefer F., Tullus K. (2020). The severity of COVID-19 in children on immunosuppressive medication. Lancet Child. Adolesc. Health..

[13] Canpolat N., Yıldırım Z.Y., Yıldız N., Taşdemir M., Göknar N., Evrengül H., Gülmez R., Aksu B., Dursun H., Özçelik G. (2022). COVID-19 in pediatric patients undergoing chronic dialysis and kidney transplantation. Eur. J. Pediatr..

[14] Kumar N., Rana R., Rana D.S., Gupta A., Sachdeva M.P. (2022). SARS-CoV-2 in Kidney Transplant Recipients: A Systematic Review. Transplantology.

[15] Kotton C.N. (2014). Immunization after kidney transplantation—What is necessary and what is safe?. Nat. Rev. Nephrol..

[16] Grupper A., Rabinowich L., Schwartz D., Schwartz I.F., Ben-Yehoyada M., Shashar M., Katchman E., Halperin T., Turner D., Goykhman Y. (2021). Reduced humoral response to mRNA SARS-CoV-2 BNT162b2 vaccine in kidney transplant recipients without prior exposure to the virus. Am. J. Transplant..

[17] Banerjee S., Dissanayake P.V., Abeyagunawardena A.S. (2016). Vaccinations in children on immunosuppressive medications for renal disease. Pediatr. Nephrol..

[18] Crane C., Phebus E., Ingulli E. (2023). Antibody response to 2- and 3-dose SARS-CoV-2 mRNA vaccination in pediatric and adolescent kidney transplant recipients. Pediatr. Nephrol..

[19] Cirillo L., Citera F., Mazzierli T., Becherucci F., Terlizzi V., Lodi L., Buti E., Romagnani P. (2022). Response to Third Dose of Vaccine Against SARS-CoV-2 in Adolescent and Young Adult Kidney Transplant Recipients. Transplantation.

[20] Kermond R.F., Ozimek-Kulik J.E., Kim S., Alexander S.I., Hahn D., Kesson A., Wood N., McCarthy H.J., Durkan A.M. (2023). Immunologic response to SARS-CoV-2 mRNA vaccination in pediatric kidney transplant recipients. Pediatr. Nephrol..

[21] Sattler A., Thumfart J., Tóth L., Schrezenmeier E., Proß V., Stahl C., Siegle J., He A., Thole L.M.L., Ludwig C. (2022). SARS-CoV2 mRNA Vaccine-Specific B-, T- and Humoral Responses in Adolescents After Kidney Transplantation. Transpl. Int..

[22] Haskin O., Ashkenazi-Hoffnung L., Ziv N., Borovitz Y., Dagan A., Levi S., Koren G., Hamdani G., Levi-Erez D., Landau D. (2021). Serological Response to the BNT162b2 COVID-19 mRNA Vaccine in Adolescent and Young Adult Kidney Transplant Recipients. Transplantation.

[23] Stich M., Di Cristanziano V., Tönshoff B., Weber L.T., Dötsch J., Rammer M.T., Rieger S., Heger E., Garbade S.F., Burgmaier K. (2023). Humoral immune response and live-virus neutralization of the SARS-CoV-2 omicron (BA.1) variant after COVID-19 mRNA vaccination in children and young adults with chronic kidney disease. Pediatr Nephrol..

[24] Gulmez R., Ozbey D., Agbas A., Aksu B., Yildiz N., Uckardes D., Saygili S., Yilmaz E.K., Yildirim Z.Y., Tasdemir M. (2023). Humoral and cellular immune response to SARS-CoV-2 mRNA BNT162b2 vaccine in pediatric kidney transplant recipients compared with dialysis patients and healthy children. Pediatr Nephrol..

[25] Benning L., Morath C., Bartenschlager M., Nusshag C., Kälble F., Buylaert M., Schaier M., Beimler J., Klein K., Grenz J. (2022). Neutralization of SARS-CoV-2 Variants of Concern in Kidney Transplant Recipients after Standard COVID-19 Vaccination. Clin. J. Am. Soc. Nephrol..

[26] Rozen-Zvi B., Yahav D., Agur T., Zingerman B., Ben-Zvi H., Atamna A., Tau N., Mashraki T., Nesher E., Rahamimov R. (2021). Antibody response to SARS-CoV-2 mRNA vaccine among kidney transplant recipients: A prospective cohort study. Clin. Microbiol. Infect..

[27] Boyarsky B.J., Werbel W.A., Avery R.K., Tobian A.A.R., Massie A.B., Segev D.L., Garonzik-Wang J.M. (2021). Antibody Response to 2-Dose SARS-CoV-2 mRNA Vaccine Series in Solid Organ Transplant Recipients. JAMA.

[28] Ślizień Z., Muchlado M., Kubanek A., Biedunkiewicz B., Renke M., Komorowska K., Dębska-Ślizień A., Tylicki L. (2022). Safety and Tolerability of mRNA COVID-19 Vaccines in Kidney Transplant Recipients. Transplant. Proc..

[29] Grupper A., Katchman E., Ben-Yehoyada M., Rabinowich L., Schwartz D., Schwartz I.F., Shashar M., Halperin T., Turner D., Goykhman Y. (2021). Kidney transplant recipients vaccinated before transplantation maintain superior humoral response to SARS-CoV-2 vaccine. Clin. Transplant..

[30] Hookham L., Lee H.C., Patel D.A., Coelho M., Giglio N., Le Doare K., Pannaraj P.S. (2022). Vaccinating Children against SARS-CoV-2: A Literature Review and Survey of International Experts to Assess Safety, Efficacy and Perceptions of Vaccine Use in Children. Vaccines.

[31] Mehrabi Nejad M.M., Shobeiri P., Dehghanbanadaki H., Tabary M., Aryannejad A., Haji Ghadery A., Shabani M., Moosaie F., SeyedAlinaghi S., Rezaei N. (2022). Seroconversion following the first, second, and third dose of SARS-CoV-2 vaccines in immunocompromised population: A systematic review and meta-analysis. Virol. J..

[32] Kreuzberger N., Hirsch C., Andreas M., Böhm L., Bröckelmann P.J., Di Cristanziano V., Golinski M., Hausinger R.I., Mellinghoff S., Lange B. (2022). Immunity after COVID-19 vaccination in people with higher risk of compromised immune status: A scoping review. Cochrane Database Syst. Rev..

[33] Van Assen S., Holvast A., Benne C.A., Posthumus M.D., Van Leeuwen M.A., Voskuyl A.E., Blom M., Risselada A.P., de Haan A., Westra J. (2010). Humoral responses after influenza vaccination are severely reduced in patients with rheumatoid arthritis treated with rituximab. Arthritis Rheum..

[34] Arad U., Tzadok S., Amir S., Mandelboim M., Mendelson E., Wigler I., Sarbagil-Maman H., Paran D., Caspi D., Elkayam O. (2011). The cellular immune response to influenza vaccination is preserved in rheumatoid arthritis patients treated with rituximab. Vaccine.

[35] Prendecki M., Willicombe M., McAdoo S.P. (2021). COVID-19 vaccination in patients with immunity-mediated kidney disease. Nat. Rev. Nephrol..

[36] Connolly C.M., Chiang T.P., Boyarsky B.J., Ruddy J.A., Teles M., Alejo J.L., Massie A., Werbel W.A., Shah A.A., Christopher-Stine L. (2022). Temporary hold of mycophenolate augments humoral response to SARS-CoV-2 vaccination in patients with rheumatic and musculoskeletal diseases: A case series. Ann. Rheum Dis..

[37] Schrezenmeier E., Rincon-Arevalo H., Jens A., Stefanski A.L., Hammett C., Osmanodja B., Koch N., Zukunft B., Beck J., Oellerich M. (2022). Temporary antimetabolite treatment hold boosts SARS-CoV-2 vaccination-specific humoral and cellular immunity in kidney transplant recipients. JCI Insight.

[38] Osmanodja B., Ronicke S., Budde K., Jens A., Hammett C., Koch N., Seelow E., Waiser J., Zukunft B., Bachmann F. (2022). Serological Response to Three, Four and Five Doses of SARS-CoV-2 Vaccine in Kidney Transplant Recipients. J. Clin. Med..

[39] Schröder D., Heinemann S., Heesen G., Klawonn F., Mikuteit M., Niewolik J., Steffens S., Behrens G., Jablonka A., Müller F. (2022). Who is pausing immunosuppressive medication for COVID-19 vaccination? Results of an exploratory observational trial. Eur. J. Med. Res..

[40] Soresina A., Moratto D., Chiarini M., Paolillo C., Baresi G., Focà E., Bezzi M., Baronio B., Giacomelli M., Badolato R. (2020). Two X-linked agammaglobulinemia patients develop pneumonia as COVID-19 manifestation but recover. Pediatr. Allergy Immunol..

[41] Quinti I., Lougaris V., Milito C., Cinetto F., Pecoraro A., Mezzaroma I., Mastroianni C.M., Turriziani O., Bondioni M.P., Filippini M. (2020). A possible role for B cells in COVID-19? Lesson from patients with agammaglobulinemia. J. Allergy Clin. Immunol..

[42] Terada K., Hagihara K., Oishi T., Miyata I., Akaike H., Ogita S., Ohno N., Ouchi K. (2017). Cellular and humoral immunity after vaccination or natural mumps infection. Pediatr. Int..

[43] Honeyman M.C., Forrest J.M., Dorman D.C. (1974). Cell-mediated immune response following natural rubella and rubella vaccination. Clin. Exp. Immunol..

[44] Page M.J., McKenzie J.E., Bossuyt P.M., Boutron I., Hoffmann T.C., Mulrow C.D., Shamseer L., Tetzlaff J.M., Akl E.A., Brennan S.E. (2021). The PRISMA 2020 statement: An updated guideline for reporting systematic reviews. BMJ.

